# Relapse activity in the chronic phase of anti-myelin-oligodendrocyte glycoprotein antibody-associated disease

**DOI:** 10.1007/s00415-021-10914-x

**Published:** 2021-11-25

**Authors:** Tetsuya Akaishi, Tatsuro Misu, Kazuo Fujihara, Toshiyuki Takahashi, Yoshiki Takai, Shuhei Nishiyama, Kimihiko Kaneko, Juichi Fujimori, Tadashi Ishii, Masashi Aoki, Ichiro Nakashima

**Affiliations:** 1 Department of Neurology, Tohoku University Graduate School of Medicine, Seiryo-machi 1-1, Aoba-ku, Sendai, Miyagi 980-8574 Japan; 2 Department of Education and Support for Regional Medicine, Tohoku University Hospital, Sendai, Japan; 3 Department of Multiple Sclerosis Therapeutics, Fukushima Medical University, Fukushima, Japan; 4 Department of Neurology, National Hospital Organization Yonezawa National Hospital, Yonezawa, Japan; 5 Department of Neurology, Tohoku Medical and Pharmaceutical University, Sendai, Japan

**Keywords:** Anti-myelin oligodendrocyte glycoprotein (MOG) antibody, MOG-antibody-associated disease (MOGAD), Neuromyelitis optica spectrum disorder (NMOSD), Relapse-free survival, Relapse prevention

## Abstract

**Objective:**

The patterns of relapse and relapse-prevention strategies for anti-myelin oligodendrocyte glycoprotein antibody-associated disease (MOGAD) are not completely investigated. We compared the patterns of relapse in later stages of MOGAD with those of anti-aquaporin-4 antibody (AQP4-Ab)-positive neuromyelitis optica spectrum disorder (NMOSD).

**Methods:**

In this observational, comparative cohort study, 66 patients with MOGAD and 90 with AQP4-Ab-positive NMOSD were enrolled. We compared the patterns of relapse and annualized relapse rates (ARRs) in the first 10 years from disease onset, stratified by relapse-prevention treatments.

**Results:**

Approximately 50% of the patients with MOGAD experienced relapses in the first 10 years. Among those not undergoing relapse-prevention treatments, ARRs in the first 5 years were slightly lower in MOGAD patients than in AQP4-Ab-positive NMOSD patients (MOGAD vs. AQP4-Ab NMOSD: 0.19 vs. 0.30; *p* = 0.0753). After 5 years, the ARR decreased in MOGAD patients (MOGAD vs. AQP4-Ab NMOSD: 0.05 vs. 0.34; *p* = 0.0001), with a 72% reduction from the first 5 years (*p* = 0.0090). Eight (61.5%) of the 13 MOGAD patients with more than 10-year follow-up from disease onset showed relapse 10 years after onset. Clustering in the timing and phenotype of attacks was observed in both disease patients. The effectiveness of long-term low-dose oral PSL for relapse prevention in patients with MOGAD has not been determined.

**Conclusions:**

The relapse risk in patients with MOGAD is generally lower than that in patients with AQP4-Ab-positive NMOSD, especially 5 years after onset. Meanwhile, relapses later than 10 years from onset are not rare in both diseases.

## Introduction

Neuromyelitis optica spectrum disorder (NMOSD) is a neurological condition, different from multiple sclerosis (MS), characterized by recurrent clinical episodes of demyelinating lesions in the central nervous system (CNS). The prevalence of NMOSD is estimated to be 0.5–10 people per 100,000 in the general population and is reportedly high among Asians than among Caucasians or African-Americans [1]. Typical clinical manifestations include optic neuritis (ON), transverse acute myelitis, area postrema syndrome, and cerebral lesions [2–4]. Until now, two disease-specific antibodies have been identified in patients with CNS lesions: anti-aquaporin-4 antibody (AQP4-Ab) and anti-myelin oligodendrocyte glycoprotein antibody (MOG-Ab) [5, 6]. In recent years, a disease concept known as MOG-Ab-associated disease (MOGAD) has emerged [7, 8]. The possible differences of MOGAD from AQP4-Ab-positive NMOSD in terms of the pathological mechanisms, outcomes, and therapeutic strategies have been gradually elucidated [9–11]. Emerging data have implied that these two diseases may be independent of each other, with different glial cell targets (oligodendrocytes for MOGAD and astrocytes for AQP4-Ab-positive NMOSD) and different rates of intrathecal synthesis of antibodies in the cerebrospinal fluid (CSF) [12, 13].

Generally, irreversible neurological sequelae after attacks are more severe in patients with AQP4-Ab-positive NMOSD than in patients with MOGAD [14, 15], although a small fraction of patients with MOGAD may also suffer from severe neurological sequelae [16]. In contrast to the progression of neurological disability in MS, neurological disability in AQP4-Ab-positive NMOSD and MOGAD is almost exclusively accrued to a sequel of attacks. Furthermore, a gradual and relapse-independent progression of disability is not typically observed in AQP4-Ab-positive NMOSD and MOGAD [17, 18]. Thus, prevention of relapse is essential in these diseases. Currently, the standard therapeutic strategies in the acute phase of AQP4-Ab-positive NMOSD and MOGAD are the administration of high-dose intravenous methylprednisolone pulse (IVMP) therapy [18–20]. After the acute phase, an indefinite period of relapse prevention with immunomodulatory medications, such as azathioprine, rituximab, eculizumab, mycophenolate mofetil, or prednisolone (PSL), is recommended for AQP4-Ab-positive NMOSD [21–25]. Meanwhile, the optimal relapse-prevention strategy in MOGAD has not been established yet. In this study, we compared the relapse pattern in MOGAD with that in AQP4-Ab-positive NMOSD and further evaluated a desirable relapse-prevention strategy for MOGAD.

## Methods

### Study design and participants

To evaluate the time-dependent relapse rates in MOGAD, we enrolled patients who had been tested for serum positivity for autoantibodies and treated at two university hospitals in Japan (Tohoku University and Tohoku Medical and Pharmaceutical University) between 2005 and 2020. To compare the time-dependency and level of relapse occurrence, AQP4-Ab-positive NMOSD patients, who were diagnosed and treated at these facilities, were also enrolled. Their medical records, clinical course data, relapse timings, laboratory test data, and imaging test data were retrospectively reviewed in September 2021. The relapse patterns in these enrolled patients were then compared between the diseases, after being stratified according to the maintenance therapy for relapse prevention from disease onset. The occurrence of relapse was defined as the development of a new neurological symptom or the worsening of an old symptom that lasted at least 24 h and occurred more than 30 days after the previous attack [26], which could be explained based on the observation of a newly appeared magnetic resonance imaging lesion. Furthermore, the clustering of the timing and phenotype of attacks in AQP4-Ab-positive NMOSD has been reported earlier [27], which may significantly influence the relapse patterns in the disease. However, the presence of the clustering of attacks in MOGAD has not been determined yet. Thus, we also evaluated the clustering of the timing and phenotype of attacks in MOGAD.

Information about the positivity of disease-specific antibodies (MOG-Ab/AQP4-Ab) in the serum and CSF, age at disease onset, age at antibody titration, sex, clinical phenotype of the onset episode (ON/acute myelitis), details of treatments for relapse prevention, and the timing of all the relapses by July 2021 were collected. MOG-Ab/AQP4-Ab positivity in the serum and CSF was confirmed using the microscopic live cell-based assay method [28, 29]. Human M23-AQP4 or human MOG expressing HEK293 cells were incubated with diluted serum samples of the patients. A secondary antibody specific for immunoglobulin G (IgG)-Fc fragment was used. Serum samples were screened at dilutions of 1:16 for AQP4-Ab and 1:128 for MOG-Ab. CSF samples were screened without diluting the samples in either of the antibodies. The treated cells were then stained with an Alexa Fluor 488-conjugated anti-human IgG Fc fragment secondary antibody (Thermo Fisher Scientific, Waltham, MA, USA). The titrations were evaluated visually semi-quantitatively using consecutive twofold end-point dilutions.

### Statistical analysis

Distributions of the quantitative data were described using median and interquartile range (that is, 25–75 percentiles). Categorical data were described using numbers and prevalence (%) for each disease group. Crude annualized relapse rates (ARRs) were calculated by dividing the total number of relapses (excluding the first attack) by the total number of person-years during the study period. Comparisons of ARRs between diseases or between time periods were performed to calculate the probability value using F-tables by assuming a Poisson process for the relapse [30]. For this, we assumed that the number of relapses in each group independently followed the Poisson distribution. When comparing the ARR values between the two groups, the crude rate ratio was calculated with its 95% confidence interval (CI). When the relapse process in the two diseases did not follow the Poisson process (for example, the relapse occurrence rate was not consistent during the follow-up period), further analyses, such as relapse-free survival analyses with Kaplan–Meier curve analysis and log-rank test, were performed to compare the time-dependent relapse between the two diseases [31]. The observations for survival were intended to be censored at the time patients commenced relapse prevention before a relapse, but no patient filled the requirements. The Cox proportional hazard regression model was applied to estimate the hazard ratio (HR) by comparing the duration of relapse-free survival between the two disease groups. Statistical significance was set at *p* < 0.05. Statistical analyses were performed using R Statistical Software (version 4.0.5; R Foundation for Statistical Computing, Vienna, Austria).

## Results

### Demographic and clinical characteristics

**Fig. 1:**
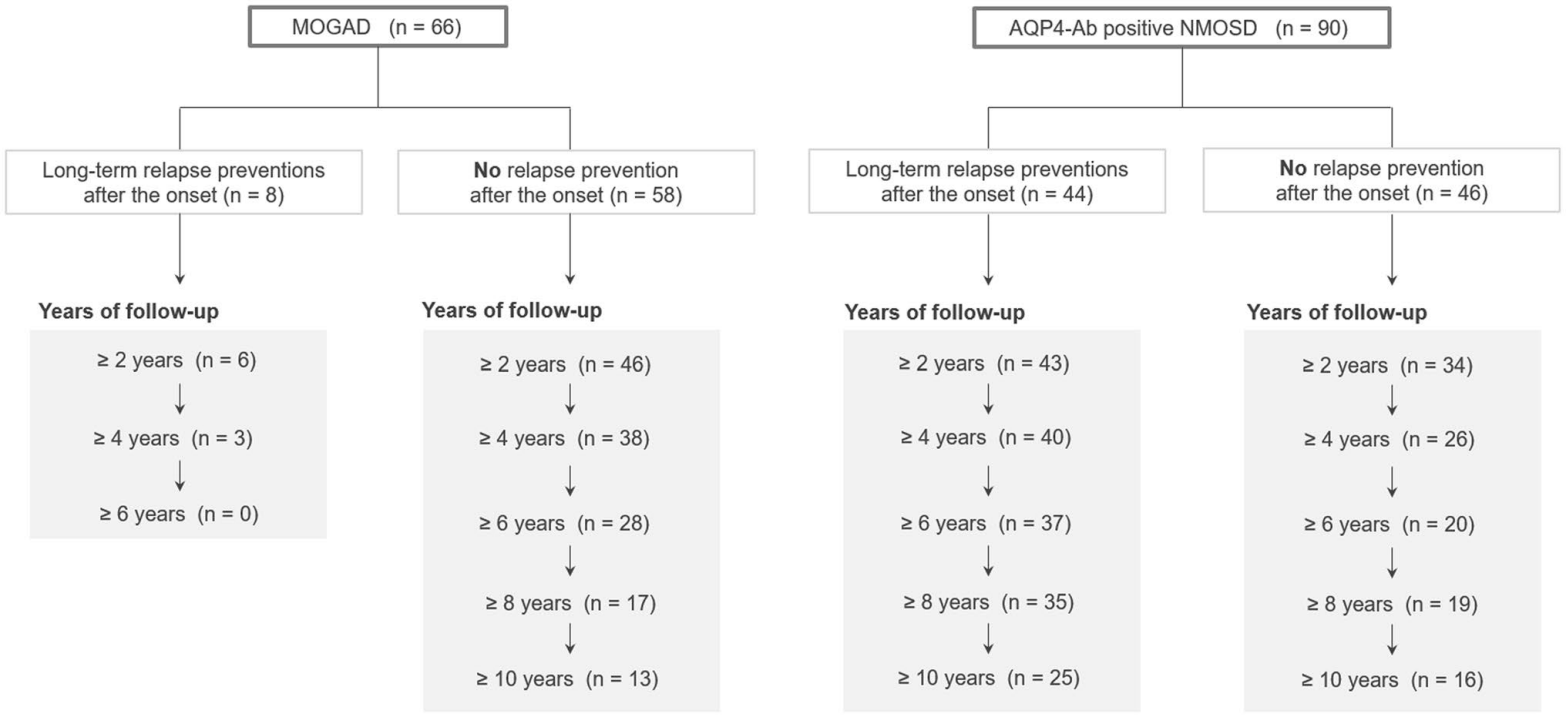
Study design flowchart. Patients with MOGAD and AQP4-Ab-positive NMOSD were enrolled in this study. Patients were divided into four groups based on the type of autoantibody and administration of long-term relapse prevention from onset. Relapse-free survival analyses were performed between the four groups for patients with different follow-up durations. *AQP4-Ab* anti-aquaporin-4 antibody, *MOGAD* anti-myelin oligodendrocyte glycoprotein antibody-associated disease, *NMOSD* neuromyelitis optica spectrum disorder, *PSL* prednisolone

A total of 66 consecutive patients with MOGAD (64 with serum MOG-Ab and two with CSF-restricted MOG-Ab) and 90 patients with serum AQP4-Ab-positive NMOSD were enrolled in this study. None of the patients had CSF-restricted AQP4-Ab. Details of the length of follow-up periods for the patients are shown in Fig. 1. Of the 66 patients with MOGAD, 62 were tested for serum and CSF MOG-Ab titers using samples acquired at the clinical onset, whereas the remaining four patients were tested using samples acquired at relapse. Furthermore, of the 90 patients with AQP4-Ab-positive NMOSD, 67 patients were tested for serum AQP4-Ab at onset, whereas the remaining 23 patients were tested with samples acquired at relapse. To prevent relapse, 8 (12.1%) of the 66 patients with MOGAD were treated with long-term oral PSL using 5–20 mg daily dose (*n* = 6), disease-modifying drugs (DMD) (*n* = 1), or a combination of both (*n* = 1) at disease onset, whereas 44 (48.9%) of the 90 patients with AQP4-Ab-positive NMOSD were treated with long-term oral PSL at the onset. In the remaining patients (58 with MOGAD and 46 with AQP4-Ab) who were not treated with long-term relapse prevention at the onset of disease, five patients with MOGAD and all 46 patients with AQP4-Ab-positive NMOSD were later treated with long-term oral PSL after the occurrence of relapse. The demographic and clinical features of the participants are summarized in the upper half of Table 1. The clinical severity, represented by the Expanded Disability Status Scale scores at the last follow-up, was significantly worse in AQP4-Ab-positive NMOSD patients than in MOGAD patients, regardless of relapse prevention. The ARR was also higher in AQP4-Ab-positive NMOSD patients than in MOGAD patients.

**Table 1:** Demographics and clinical features of the participants

	MOGAD		AQP4-Ab NMOSD	
	With relapse prevention from onset	No relapse prevention	With relapse prevention from onset	No relapse prevention
*n*	8	58	44	46
Male:female (*n*)	3:5	25:33	4:40	0:46
Onset age (years)*	40 (32–50)	34 (20–48)	51 (38–58)	38 (33–47)
Follow-up period (years)*	3.0 (2.5–4.0)	5.0 (3.0–10.0)^a^	10.0 (8.0–14.0)	4.0 (1.0–11.0)^a^
ON at onset (*n* [%])	4/8 (50.0%)	37/58 (63.8%)	20/44 (45.5%)	19/46 (41.3%)
EDSS at the last follow-up*	0.0 (0.0–0.0)	0.0 (0.0–0.0)	4.0 (3.0–6.0)	5.0 (3.5–6.5)
Negative conversion of serum MOG-Ab within 24 months, *n* (%)	4/7 (57.1%)	5/11 (45.5%)	–	–
Acute myelitis at onset (*n* [%])	4/8 (50.0%)	8/58 (13.8%)	24/44 (54.5%)	20/46 (43.5%)
IVMP therapy at onset (*n* [%])	7/8 (87.5%)	43/58 (74.1%)	41/44 (93.2%)	20/46 (43.5%)
Annualized relapse rate	0.084	0.109	0.211	0.304
Number of patients having relapses in earlier disease stages, *n* (%)				
In first 2 years (months 0–23)	1/6 (16.7%)	13/46 (28.3%)	13/43 (30.2%)	27/45 (60.0%)
In first 4 years (months 0–47)	2/3 (66.7%)	19/38 (50.0%)	18/40 (45.0%)	38/45 (84.4%)
In first 5 years (months 0–59)	0/1 (0.0%)	17/30 (56.7%)	17/38 (44.7%)	17/22 (77.3%)
In first 10 years (months 0–119)	0/0	7/13 (53.8%)	14/25 (56.0%)	12/16 (75.0%)
Number of patients having relapses in later disease stages, *n* (%)				
In second 2 years (months 24–47)	2/3 (66.7%)	9/38 (23.7%)	14/40 (35.0%)	16/26 (61.5%)
In second 4 years (months 48–95)	0/0	3/17 (17.6%)	12/35 (34.3%)	13/19 (68.4%)
In second 5 years (months 60–119)	0/0	2/13 (15.4%)	7/25 (28.0%)	10/16 (62.5%)

Data regarding relapse occurrence in the groups without relapse-prevention treatment after onset are for the period free of any relapse treatments

*AQP4-Ab* anti-aquaporin-4 autoantibody, *EDSS* Expanded Disability Status Scale, *IVMP* intravenous methylprednisolone pulse therapy, *MOGAD* anti-myelin oligodendrocyte glycoprotein antibody-associated disease, *NMOSD* neuromyelitis optica spectrum disorder, *PSL* prednisolone

*Median and interquartile range (that is, 25–75 percentiles)

^a^Follow-up period without long-term oral PSL for relapse prevention

### ARR during periods without relapse prevention

The time course of occurrence of relapse in the first 10 years from onset is shown for the two diseases in the lower half of Table 1. During the period from onset without relapse prevention, the crude ARR was lower in MOGAD patients than in AQP4-Ab-positive NMOSD patients (0.11 vs. 0.30, *p* < 0.0001). The ARRs in the first 2 years from onset were not different between the two disease patients (0.23 in MOGAD vs. 0.26 in AQP4-Ab-positive NMOSD; *p* = 0.7255), with a crude rate ratio of 0.88 (95% CI 0.46–1.74). The ARRs in the first 5 years were only slightly lower in MOGAD patients than in AQP4-Ab-positive NMOSD patients (0.19 vs. 0.30, *p* = 0.0753), with a crude rate ratio of 0.64 (95% CI 0.38–1.05).

Next, to confirm the time-dependent ARR decrease in MOGAD patients, the ARRs in earlier and later disease stages during the periods without relapse prevention were compared for each disease. In AQP4-Ab-positive NMOSD patients, the ARR in 60–119 months did not differ from that in 0–59 months (0.34 vs. 0.30; *p* = 0.6745), with a rate ratio of 1.13 (95% CI 0.65–1.90). In contrast, the ARR in MOGAD patients significantly decreased in 60–119 months from that in 0–59 months (0.05 vs. 0.19, *p* = 0.0090), with a crude rate ratio of 0.28 (95% CI 0.06–0.74).

### Relapse-free survival for 10 years from onset

**Fig. 2:**
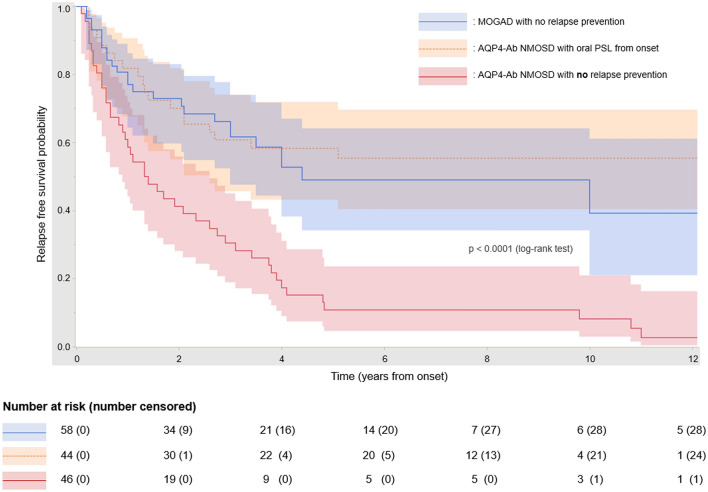
Relapse-free survival analysis by disease groups. Kaplan–Meier curves for the relapse-free survival period and the results of the log-rank test in patients with MOGAD and AQP4-Ab-positive NMOSD are shown. In patients who did not receive relapse-prevention treatments, the relapse activity was higher in AQP4-Ab-positive NMOSD than in MOGAD, and patients with MOGAD had a higher chance of remaining relapse-free. The numbers in the table below the Kaplan–Meier curves represent the numbers of patients who were followed up and the numbers censored at each time point in each of the disease groups

Kaplan–Meier curves for the relapse-free survival analysis of patients with MOGAD and AQP4-Ab-positive NMOSD, who were not treated for long-term relapse prevention, are shown in Fig. 2. The Kaplan–Meier curve for patients with AQP4-Ab-positive NMOSD, who were treated for long-term relapse prevention from the onset, is also shown. No curve was depicted for patients with MOGAD who were treated for long-term relapse prevention from the onset, as the sample size was small (less than ten patients). The relapse activity from onset was significantly higher in untreated AQP4-Ab-positive NMOSD patients than in untreated MOGAD patients (*p* < 0.0001, log-rank test), with an estimated unadjusted HR of 0.35 (95% CI 0.21–0.57), obtained by applying the Cox proportional hazard regression model. Consequently, untreated patients with MOGAD had a higher chance of remaining relapse-free compared to those with AQP4-Ab-positive NMOSD. Furthermore, a significant effect of relapse prevention with long-term oral PSL was observed in patients with AQP4-Ab-positive NMOSD (unadjusted HR, 0.30; 95% CI 0.18–0.52; *p* < 0.0001).

### Relapse-free survival in the earlier and later disease stages

**Fig. 3:**
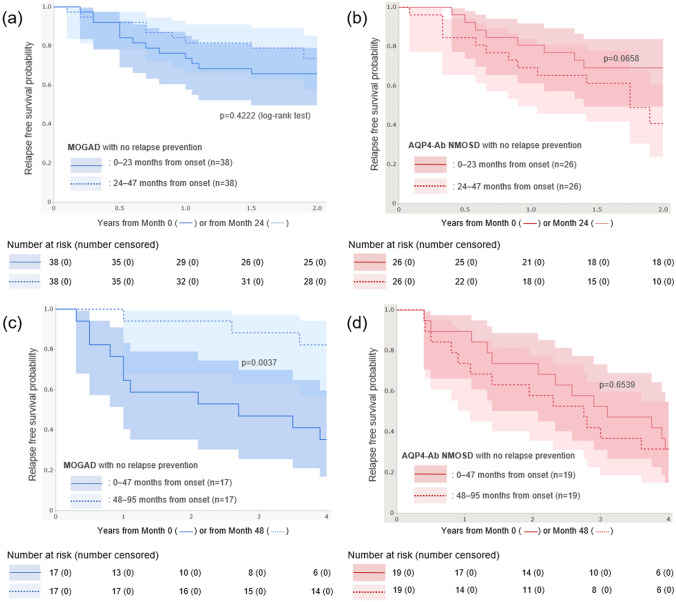
Relapse-free survival analysis based on the time from onset. Kaplan–Meier curves for relapse-free survival in the first 2 years and in the following 2 years are depicted for MOGAD (**a**) and AQP4-Ab-positive NMOSD patients (**b**). Relapse-free survival time did not differ between the first 2 years and the next 2 years for both diseases. Next, Kaplan–Meier curves for the first 4 years and the following 4 years are depicted for MOGAD (**c**) and AQP4-Ab-positive NMOSD patients (**d**). Relapse activity decreased in the later stages after 4 years from disease onset in MOGAD patients, whereas such time-dependent decrease in relapse activity was not observed in AQP4-Ab-positive NMOSD patients. *AQP4-Ab* anti-aquaporin-4 antibody, *NMOSD* neuromyelitis optica spectrum disorder, *MOGAD* anti-myelin oligodendrocyte glycoprotein antibody-associated disease

To determine the presence of time-dependent changes in the relapse activity of the two diseases, relapse-free survival analyses were performed after dividing the follow-up period into early and later disease stages with different cut-off time points. Patients not undergoing long-term relapse-prevention treatments during the periods of interest were included in the analysis. The Kaplan–Meier curves for relapse-free survival in the first 2 years from onset and in the following 2 years are shown in Fig. 3a and b. It was observed that in both the diseases, the relapse activity did not differ between the earlier and later disease stages. The Kaplan–Meier curves for relapse-free survival in the first 4 years from onset and in the following 4 years are depicted in Fig. 3c and d. The relapse activity in later disease stages decreased in MOGAD patients but not in AQP4-Ab-positive NMOSD patients. By applying a Cox proportional hazard model, the HR for relapse occurrence after 4 years from onset in MOGAD patients, as compared to that in the first 4 years, was estimated to be 0.21 (95% CI 0.06–0.77).

### Relapses after 10 years from disease onset

Thirteen MOGAD patients not undergoing relapse-prevention treatments were followed up for more than 10 years from the first neurological episode without any relapse-prevention treatments, with a total of 141 years of follow-up 10 years after the onset. Among them, eight patients (61.5%) had a total of nine relapses 10 years after the first neurological episode, with the time period of relapse ranging between 10 and 46 years from the onset. In AQP4-Ab-positive NMOSD, 12 patients not undergoing relapse-prevention treatments were followed up for more than 10 years from the first episode without any relapse-prevention treatments, with a total of 77.3 follow-up years. Among them, eight patients (66.7%) had a total of 15 relapses 10 years after the first neurological episode, with the time period of relapse ranging from 10 to 17 years from onset. The calculated crude rate ratio after 10 years between MOGAD and AQP4-Ab-positive NMOSD patients was 0.33 (95% CI 0.13–0.73), which was significantly different between the two diseases (*p* = 0.0069). These results suggest that in both MOGAD and AQP4-Ab-positive NMOSD patients not undergoing relapse-prevention treatments, relapses are not rare 10 years after disease onset, although relapse activity was observed to be lower in MOGAD.

### Clustering in the timing of attacks and phenotypes

**Fig. 4:**
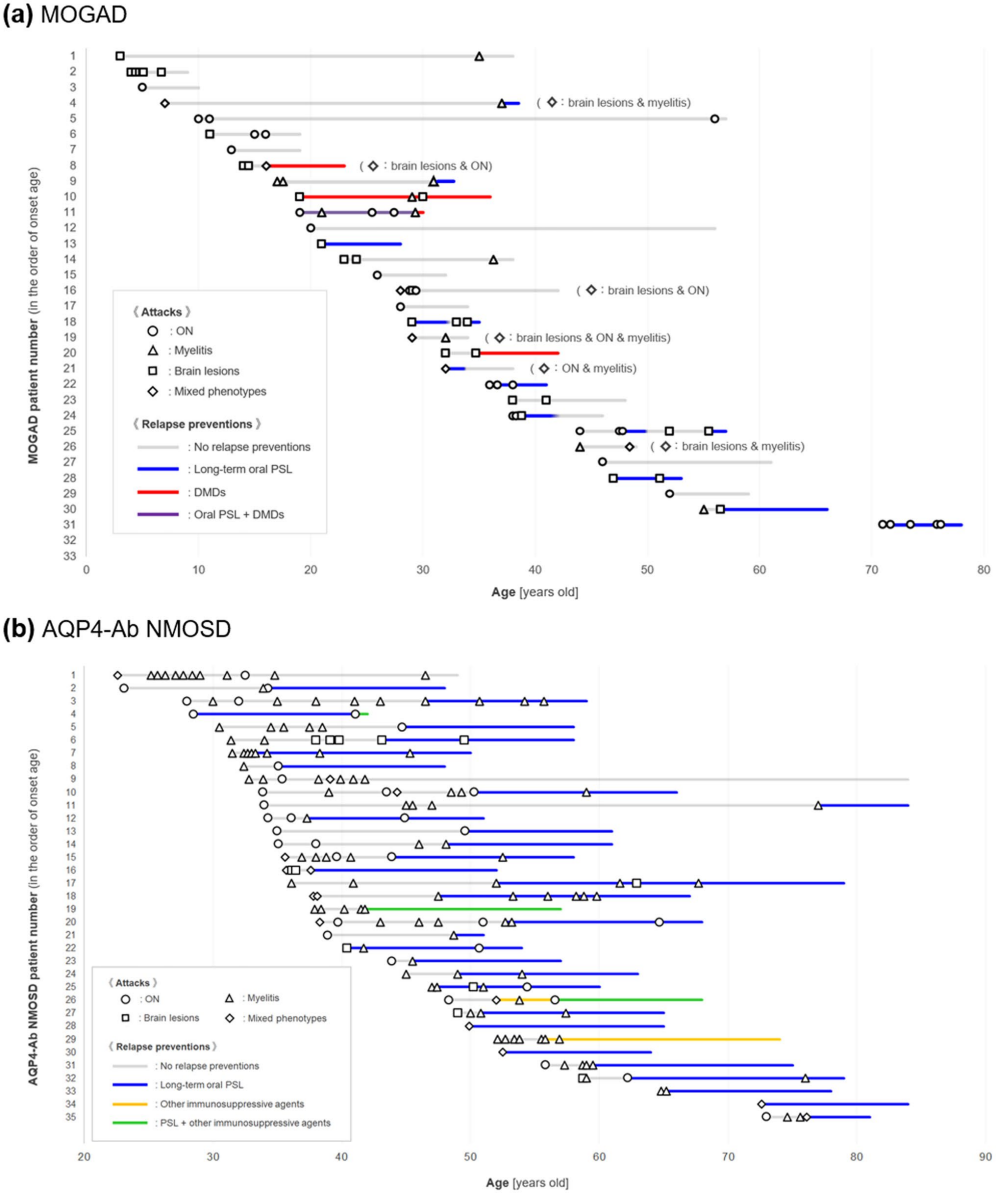
Patterns of attack occurrence and phenotypes. The time course of attack occurrence and clinical phenotypes of the 33 patients with MOGAD (**a**) and 35 patients with AQP4-Ab-positive NMOSD (**b**), who were followed up for ≥ 5 years and with complete detailed attack information are shown. Clustering of attacks within 2–3 years after the first clinical episode was observed in some of the patients with MOGAD, which could have partially contributed to the observed difference in relapse frequency between the earlier and later disease stages of MOGAD. In addition to the timing of attack occurrence, phenotypic clustering was also observed in both diseases, especially in tandem attacks with intervals of < 2 years. *AQP4-Ab* anti-aquaporin-4 antibody, *DMDs* disease-modifying drugs, *MOGAD* anti-myelin oligodendrocyte glycoprotein antibody-associated disease, *NMOSD* neuromyelitis optica spectrum disorder, *ON* optic neuritis, *PSL* prednisolone

To visually evaluate the existence of clustering of the timing of attacks and phenotypes, the clinical course of 33 MOGAD patients with a follow-up of 5 or more years is depicted in Fig. 4a. In some patients, clustering of the timing of the attacks was apparent, especially in the first 2–3 years from disease onset. Such clustering of attacks in MOGAD could have resulted in the aforementioned difference in relapse frequency in the earlier and later stages of the disease. To compare clustering of attacks between the two diseases, the clinical course of the 35 patients with AQP4-Ab NMOSD with a follow-up of 5 or more years is depicted in Fig. 4b, along with detailed information about the attack. Clustering of attacks was also apparent in AQP4-Ab NMOSD patients, which was more pronounced than that in MOGAD patients.

Furthermore, the phenotypic clustering of attacks in MOGAD was evaluated. Based on the findings in Fig. 4a, tandem attacks were likely to exhibit the same attack phenotypes. To statistically evaluate the impact of attack intervals on phenotypic clustering, the patterns of similarity in tandem attack phenotypes were evaluated according to the attack intervals. Of the 66 patients with MOGAD, there were 54 tandem attacks in 29 patients. Among the 54 tandem attacks, 39 patients (72.2%, 95% CI 59.1–82.4%) had the same attack phenotypes. Among 48 tandem attacks with intervals of < 5 years, 36 patients (75.0%, 95% CI 61.2–85.1%) had the same attack phenotypes. Furthermore, among 33 tandem attacks with an interval of < 2 years, 26 patients (78.8%, 95% CI 62.3–89.3%) had the same attack phenotypes.

### Long-term low-dose oral PSL for suppressing relapses in MOGAD

**Fig. 5:**
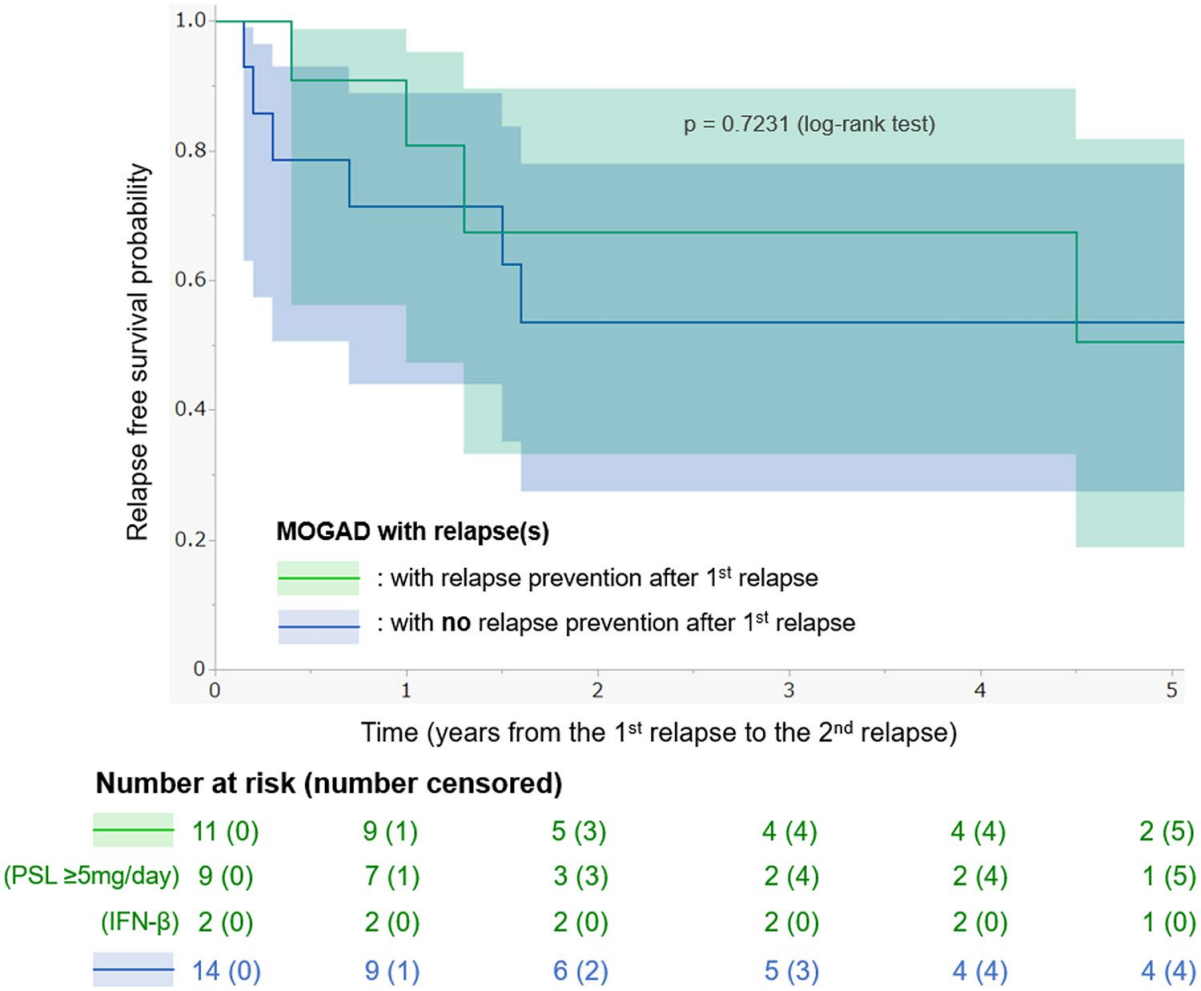
Relapse-free survival analysis by relapse prevention in MOGAD patients. To evaluate the effectiveness of long-term low-dose (that is, 5–10 mg/day for many years without cessation) oral PSL maintenance therapy for suppressing relapses in MOGAD, Kaplan–Meier curves for relapse-free survival after the first relapse by the types of relapse prevention after the first relapse are depicted. Eleven patients were treated with long-term relapse prevention (nine with oral PSL maintenance therapy and two with IFN-β autoinjection), and the other 14 patients did not undergo relapse-prevention treatments. The results could not determine the effectiveness of long-term low-dose oral PSL in suppressing relapses in MOGAD. *IFN-β* interferon-β, *MOGAD* anti-myelin oligodendrocyte glycoprotein antibody-associated disease, *PSL* prednisolone

Finally, the effectiveness of long-term low-dose oral PSL maintenance therapy with ≥ 5 mg daily dose to suppress the occurrence of relapse in MOGAD patients was evaluated. The Kaplan–Meier curves for relapse-free survival after the first relapse in MOGAD patients with at least one relapse, divided by the relapse-prevention treatments administered after the first relapse (nine with long-term PSL, two with interferon-β, and 14 with no relapse prevention), are depicted in Fig. 5. The results failed to confirm the effectiveness of long-term low-dose oral PSL in suppressing relapse in MOGAD patients.

To further investigate the impact of long-term relapse prevention with low-dose (that is, 5–10 mg/day) oral PSL or DMDs on relapse activity in MOGAD patients, the ARR data after the first relapse (that is, second clinical episode) were aggregated and compared between the periods with relapse prevention and periods without relapse prevention. The ARR during the periods with relapse prevention after the first relapse was 0.131 (10 relapses in 76.1 follow-up years from 19 patients) and the ARR during the periods with no relapse prevention was 0.127 (15 relapses in 118.4 follow-up years from 16 patients). The calculated crude rate ratio between the periods with relapse prevention and periods without relapse prevention was 1.03 (95% CI 0.44–2.26), which was not significant.

## Discussion

In this study, the time-dependent relapse occurrence was evaluated and compared between patients with MOGAD and AQP4-Ab-positive NMOSD. Based on the results of the relapse-free survival analyses, the two diseases showed different patterns in the accumulated rates of relapse occurrence in a time-dependent manner, irrespective of the clinical phenotype of the onset episode. Relapse occurrence risk was lower in MOGAD patients than in AQP4-Ab-positive NMOSD patients, especially in the later disease stages, 5 years after the first clinical episode. The decrease in relapse activity 5 years after onset was not confirmed in AQP4-Ab-positive NMOSD patients who were not given long-term relapse-prevention treatment. Our finding suggests that different approaches are applicable for long-term relapse prevention in MOGAD and AQP4-Ab-positive NMOSD patients. As suggested by previous studies [32–34], it is acceptable to indefinitely continue relapse-prevention treatment in all patients with AQP4-Ab-positive NMOSD from the disease onset. In MOGAD, the initiation of long-term relapse prevention after the first clinical episode may be spared unless the irreversible neurological sequelae after the first attack were severe, as the expected relapse activity and the levels of irreversible clinical sequelae in MOGAD patients are generally lower than those in AQP4-Ab-positive NMOSD patients. Currently, there is no evidence whether a short-term (that is, 6–12 months) post-pulse oral corticosteroids in all MOGAD patients are needed. Judged from the generally good response of MOGAD patients to IVMP therapy as acute treatment, it can be estimated that steroids would efficiently influence the process of the development of CNS lesions in MOGAD. Thus, a short-term post-pulse oral corticosteroid therapy as current practice seems to be potentially beneficial and should be or may be considered after the first episode. In any cases, long-term relapse prevention should be considered after the first relapse (i.e., the second clinical episode). The long-term relapse-prevention strategy (e.g., oral PSL daily dose, duration of treatment, and combination therapy with other steroid-sparing agents or DMDs) in MOGAD patients should be carefully judged on a patient-by-patient basis, based on the achievement status of MOG-Ab negative seroconversion, the preceding relapse frequency, and the level of neurological sequelae in the course of the disease of the patient.

This study demonstrated that relapse activity may decrease in MOGAD patients after 5 years from the onset. Considering this point, a relapse-free period of 5 years without relapse prevention might be a conceivable time point to temporarily cease the regular follow-up. Meanwhile, this study also showed that more than half of the patients who were not administered relapse-prevention treatment eventually experienced relapses 10 years from the onset. Thus, even after the aforementioned desirable follow-up period, the patients with MOGAD should be informed by physicians about their expected subsequent clinical course with possible relapses long after the last attack, and should be advised to consult physicians immediately when they notice emerging neurological symptoms suggestive of relapses.

Although the relapse activity and neurological sequelae in MOGAD patients are generally better than those in AQP4-Ab-positive NMOSD patients, some patients with MOGAD may experience severe irreversible neurological sequelae (for example, visual loss after ON or paraparesis after myelitis) after clinical attacks, regardless of appropriate administration of the acute treatment. Future research is needed to determine whether these rare cases of MOGAD with frequent relapses and irreversible severe neurological sequelae, as compared to MOGAD cases with mild manifestations, should be aggressively treated by longer and more effective relapse-prevention treatments.

There were patients with MOGAD who had their clinical onset in childhood and had relapses more than 30 years after the last attack, when the serum MOG-Ab positivity was confirmed. In these patients, a problem remains about whether their first clinical episode in childhood was really associated with MOGAD, as the serum MOG-Ab was not checked upon the first episode. However, judged from their onset ages in childhood (which are not so popular in MS or AQP4-Ab-positive NMOSD), good recovery in neurological disturbances after the first attack, types of the first clinical episode, and distributions of the CNS lesions of the first attack, it can be inferred that their first clinical episodes in their childhood could be related to the presence of MOG-Ab, and might be able to be included in the series of attacks related to MOGAD.

Finally, this study failed to statistically determine the effectiveness of long-term oral PSL maintenance therapy with or without DMDs for relapse prevention in the total cohort of the MOGAD patients. However, it should be emphasized that this does not mean that oral PSL and other steroid-sparing agents are ineffective for suppressing relapses in MOGAD [35]. This study enrolled relatively small numbers of MOGAD patients, and the cohort size could be insufficient to statistically determine the effectiveness of the treatment. Also, by evaluating the clinical course in each patient, there were not a few patients with MOGAD for whom the oral PSL maintenance therapy with ≥ 5 mg daily dose seemed to have effectively prevented relapses. As mentioned above, oral PSL and other steroid-sparing agents would be potentially beneficial for suppressing disease activity and relapses in MOGAD patients, and may be actively considered as relapse prevention, especially for those with relapses or severe irreversible neurological sequelae.

This study has several limitations. First, the evaluated cohort was exclusively comprised of patients of Asian ethnicity. Thus, the generalizability of the results to other patient cohorts with different ethnicities is uncertain. Second, the number of patients with MOGAD who were continuously treated with long-term PSL for relapse prevention from onset (*n* = 8) or the number of patients with MOGAD followed up for more than 10 years (*n* = 13) was relatively small for determining the relapse activity in the later disease stages of MOGAD. Third, this study used anti-human IgG Fc-segment secondary antibodies, not anti-human IgG1-specific secondary antibodies. This might have resulted in a lower specificity for the detection of MOG-Ab [36]. Finally, this study had some methodological weaknesses, such as its retrospective nature, various and variable types of the used relapse-prevention treatments, and the non-standardized follow-up periods. To draw firm conclusions with high evidence levels, a prospective randomized comparative study may be needed in the future.

In conclusion, relapse activity in patients with MOGAD is generally lower than that in patients with AQP4-Ab-positive NMOSD. The relapse activity in MOGAD further decreased 5 years after the first clinical episode, even without relapse prevention. However, for both the diseases, more than half of the patients without relapse prevention experienced relapses 10 years after onset. A relapse-prevention strategy for each patient with MOGAD should be considered on a patient-by-patient basis, based on achievement of MOG-Ab negative seroconversion, relapse activity, or clinical severity. However, long-term follow-up with careful perseverance to counter possible relapses may be needed in all patients.

## Data Availability

Anonymized data will be provided by the authors upon reasonable request from qualified clinicians and researchers within 5 years of publication.

## Abbreviations

AQP4-Ab: Anti-aquaporin-4 antibody
ARR: Annualized relapse rate
HR: Hazard ratio
IVMP: High-dose intravenous methylprednisolone
MOGAD: Anti-myelin oligodendrocyte glycoprotein antibody-associated disease
MOG-Ab: Anti-myelin oligodendrocyte glycoprotein antibody
MS: Multiple sclerosis
NMOSD: Neuromyelitis optica spectrum disorder
ON: Optic neuritis
